# TRAF7 in signaling and disease: emerging mechanisms and clinical implications

**DOI:** 10.1186/s10020-025-01401-4

**Published:** 2025-12-11

**Authors:** Albert Orock, Jeffrey A. Zuccato, Khanh Phan, Yufeng Liu, Jennifer Ihuoma, Sherwin Tavakol, Alla V. Tsytsykova, Erdyni N. Tsitsikov, Stefano Tarantini, Anthony C. Johnson, Ian F. Dunn

**Affiliations:** 1 Department of Neurosurgery, University of Oklahoma Health Sciences Center, Oklahoma City, OK USA; 2 Department of Neurosurgery, Massachusetts General Hospital, Harvard Medical School, Boston, MA USA

**Keywords:** Tumor necrosis factor receptor-associated factors (TRAFs), TRAF7 mutation, TRAF7 syndrome, Meningioma, Diagnostic biomarker, Prognostic biomarker

## Abstract

Tumor necrosis factor receptor-associated factors (TRAFs) are a family of 7 signaling proteins that have regulatory roles in multiple fundamental cellular processes, including immunity, inflammation, apoptosis, permeability, and cell proliferation. TRAF7 is the most recently described with unique features distinguishing it from other TRAFs. It is an E3 ubiquitin ligase that activates MEKK3 and KLF2/4 signaling, inhibits MEK1/2 and c-Myb along with an NF-κβ-modulator, and stabilizes VE-cadherins in cell junctions. Germline mutations in *TRAF7* lead to developmental delays and the dysmorphic features associated with TRAF7 syndrome. Somatic *TRAF7* mutations are associated with subsets of meningiomas, mesotheliomas, and perineuriomas. Additionally, TRAF7 altered expression is associated with poorer prognoses in hepatocellular carcinoma, breast cancer, and prostate cancer. This review comprehensively describes the physiological roles of TRAF7 and the pathophysiology of clinical conditions with TRAF7 alterations. We highlight important directions for future work to improve our understanding of the mechanisms underlying TRAF7 related disease, identify prognostic biomarkers that help guide clinical decision making, and potentially identify novel therapeutic targets to expand our treatment options for these patients.

## Background

**Fig. 1:**
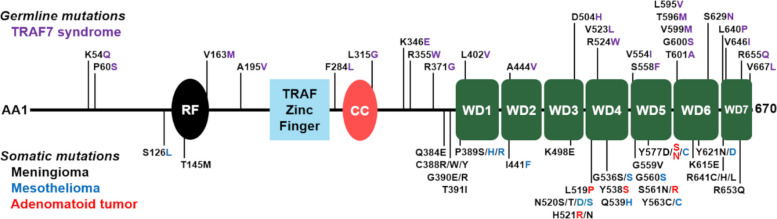
Map of TRAF7 protein highlighting sites of point mutations in germline or somatic disease. Amino acid (AA) number is on the central horizontal line starting at the N-terminus (AA1) and ending at the final amino acid (670). Key regions within TRAF7 are indicated at the approximate location: ring finger (RF), TRAF zinc finger, coiled coil domain (CC), and tryptophan-aspartic acid (W-D) dipeptide (WD40) repeats 1–7. Point mutations are indicated by the single-character abbreviation for the wild-type amino acid-site-mutated amino acid (X#Y) with vertical lines extending upwards or downwards from the protein sequence to indicate location. Germline mutations identified in TRAF7 syndrome are indicated with the mutant amino acid color coded in purple and linear location indicators extending upward. Somatic mutations are shown with mutated amino acids color-coded as black for meningioma, blue for well-differentiated mesothelioma, and red for genital tract adenomatoid tumors, with linear location indicators extending downwards

TRAF7 is a member of the tumor necrosis factor (TNF) receptor-associated factor (TRAF) family of proteins (Zotti et al. 2012). The TRAF family consists of modular regulatory adapter proteins mainly localized in the cytoplasm, which modulate signal transduction downstream of receptor complexes (Mishra-Gorur et al. 2023; Park 2018). While the functions of TRAF1-6 are well characterized, much less is known about TRAF7, the most recent to be identified. Similar to the other TRAF proteins, TRAF7 contains the characteristic N-terminal RING (Really Interesting New Gene) finger and zinc finger domains (Palma-Milla et al. 2024). TRAF7 does not have the conserved C-terminal TRAF domain typically involved in protein and receptor binding (typically found in other TRAF proteins) and instead carries seven tryptophan-aspartic acid (W-D) dipeptide (WD40) repeats at the C-terminal, leading to it also being classified as RFWD1 of the RING finger and WD domain (RFWD) family of proteins (Tsitsikov et al. 2023) (Fig. 1).

The TRAF domain in TRAF1-6 functions as both a scaffolding protein and E3 ubiquitin ligase (Park 2018). Without the TRAF domain, TRAF7 does not have any scaffolding ability, however, TRAF7 still functions as an E3 ubiquitin ligase involved in a range of important signaling pathways. The presence of the WD40 repeats introduces new protein interaction sites allowing TRAF7 to also act as a kinase, a function not performed by the other TRAF proteins.

TRAF7 was initially identified structurally as part of a complex of proteins together with mitogen-activated protein kinase kinase kinase 3 (MAP3K3/MEKK3), mitogen-activated protein kinase kinase 5 (MAP2K5/MEK5), serine/threonine-protein kinase MARK2 (EMK1), prefoldin 2 (PFDN2), heat shock protein 70 (HSP70), and others (Bouwmeester et al. 2004; Xu et al. 2004). These MAPK complexes respond to a variety of extracellular stimuli and transduce them to intracellular signaling cascades. TRAF7 also interacts with CYLD lysine 63 deubiquitinase (CYLD), a regulator of TRAF7; NF-kappa-B essential modulator (NEMO) (Zotti et al. 2011), regulating nuclear factor kappa-light-chain-enhancer of activated B cells (NF-κβ) immune-related pathways; and roundabout homolog 4 (ROBO4), regulating vascular permeability (Yoshida et al. 2005; Shirakura, et al. 2019). TRAF7 is also essential for the formation of blood vessels (Tsitsikov et al. 2023).

*TRAF7* mutations typically disrupt E3 ligase catalytic activity, which has been shown to alter its interaction with MAPK and thereby influence downstream MAPK signaling. *TRAF7* mutations also lead to activation of Ras GTPase signaling (Sahm et al. 2025), while additionally influencing the activation of NF-κβ (Shirakura, et al. 2019) and the regulation of inflammation-induced endothelial cell hyperpermeability (Bouwmeester et al. 2004). *TRAF7* mutations can also influence the activation of shear stress-responsive transcription factors KLF2 and KLF4 by altering MAPK signaling pathways involved in the vascular response to shear stress (Sohn et al. 2005).

Overall, *TRAF7* alterations are associated with an expanding portfolio of pathophysiological conditions due to their impact on various regulatory signaling pathways. Germline mutations in *TRAF7* are associated with a syndrome characterized by developmental delay, congenital heart disease, limb anomalies, and dysmorphic features (Palma-Milla et al. 2024; Tokita et al. 2018). Somatic *TRAF7* mutations, when they occur in conjunction with mutations in the *KLF4* gene, are associated with secretory meningioma development (Mishra-Gorur et al. 2023; Reuss et al. 2013). Increased *TRAF7* expression inhibits the expression of *TP53* in breast cancer and hepatocellular carcinoma, and is associated with tumor progression (Zhang et al. 2021).

In this review, we comprehensively outline the multiple functional roles of TRAF7, describe the pathophysiology of *TRAF7* mutations and related diseases, and highlight important avenues of TRAF7 investigation. Here we present the main signaling pathways and associated effects involving TRAF7. We examined how alterations of TRAF7 expression in these pathways lead to pathophysiology in a range of disorders from tumors to development disease. We review how TRAF7 may function as an oncogene or a tumor suppressor, depending on the condition and underlying pathophysiology. Finally, we discuss the challenges, limitations, and potential avenues for targeted treatment and novel biomarkers for TRAF7 disorders in preclinical and clinical studies.

### Main signaling pathways and functional roles of TRAF7

As the most recent TRAF protein to be identified, the functions of TRAF7 are incompletely characterized. However, its involvement in several essential cellular pathways has been well described. TRAF7 has two important structural domains that each relate to different aspects of its function, the conserved N-terminal RING domain that all TRAF proteins have, and the WD40 repeats that are present only in TRAF7 (see Fig. 1). Activation at the WD40 domain induces kinase activity, while activation of the ring finger domain leads to E3 ubiquitin ligase and apoptotic activity. In this way, these two domains enable TRAF7 to function either as a cytoplasmic regulatory protein and/or as a signal transducer by interacting with kinases and influencing gene transcription. In some instances *TRAF7* can act as a tumor suppressor gene and loss of function mutations precipitate cancer development (meningiomas) (Ye et al. 2023). In other cases, TRAF7 acts as an oncogene and over expression is associated with cancer (hepatocellular carcinoma) (Zhang et al. 2021). Overall, four main downstream processes occur following TRAF7 activation: kinase activation, ubiquitination, SUMOylation, and protein stabilization or destabilization (Xu et al. 2004).

### TRAF7-mediated kinase activity regulation

**Fig. 2:**
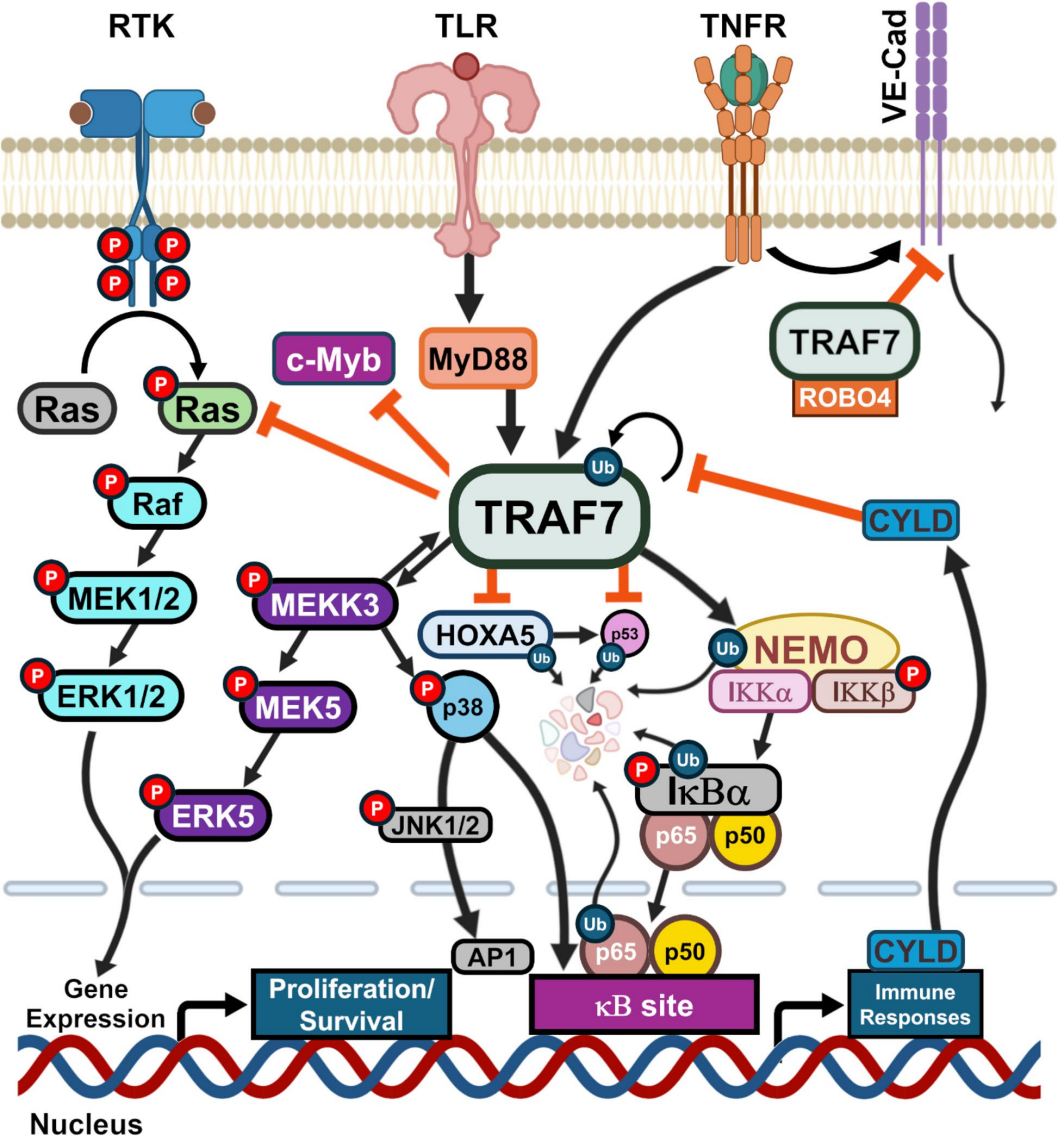
Simplified schematic of primary TRAF7 interactions with intracellular signaling cascades. (Left) In response to receptor tyrosine kinase (RTK) activation, TRAF7 can inhibit the family of rat sarcoma virus (Ras)-GTPases to regulate the Ras-Raf-MEK1/2-ERK1/2 pathway that leads to cell proliferation and survival. TRAF7 has also been found to modulate MEKK3-MEK5-ERK5 signaling. TRAF7 disrupts the activity of the proto-oncogenic transcription factor c-Myb by inducing SUMOylation. (Middle) TRAF7 participates in canonical nuclear factor kappa-light-chain-enhancer of activated B cells (NF-κβ) signaling in response to toll-like receptor (TLR) or tumor necrosis factor α receptor (TNFR) activation via increasing ubiquitination of NEMO, Iκβ, and p65, with CYLD inhibiting TRAF7’s effects. TRAF7-induced MEKK3 activation can also phosphorylate p38, which can then signal through JNK1/2 and/or the κβ site. Further, TRAF7 can ubiquitinate both the transcription factor HOXA5 and one of its targets, P53. (Right) TRAF7 interaction with roundabout guidance receptor 4 (ROBO4) can inhibit hyperpermeability induced by TNFR activation by maintaining the localization of vascular endothelial (VE)-cadherin function at tight junctions. Protein degradation after ubiquitination is illustrated as the clustered shapes in the center. Raf, rapidly accelerated fibrosarcoma; MEK1/2, mitogen-activated protein kinase 1/2; ERK1/2, extracellular signal-related kinase 1/2; MEKK3, mitogen-activated protein kinase kinase kinase 3; MEK5, mitogen-activated protein kinase 5; ERK5, extracellular signal-related kinase 5; c-Myb, MYB proto-oncogene (transcription factor), MyD88, myeloid differentiation primary response 88; IKKα, inhibitor of nuclear factor kappa-B kinase subunit alpha; IKKβ, inhibitor of nuclear factor kappa-B kinase subunit beta; NEMO, NF-kappa-B essential modulator;Iκβα, inhibitor of nuclear factor kappa B; p50, NF-κβ1. p65, v-rel avian reticuloendotheliosis viral oncogene homolog A; p38, p38 mitogen-activated protein kinase; CYLD, CYLD lysine 63 deubiquitinase; HOXA5, homeobox A5; p53, tumor protein 53; JNK1/2, c-Jun N-terminal kinase 1/2; AP-1, activator protein 1; P, phosphate; Ub, ubiquitin. The graphics were designed on BioRender

One major influence of TRAF7 on intracellular signaling relates to the regulation of kinase activity which mediate cellular responses to external stimuli. TRAF7 complexes with MEKK3 and other MAPKs, regulating their kinase activity. The MAPK signal transduction pathways which include MEKK3 are activated by extracellular signals including cytokines, osmotic stress, and vascular shear stress (Tsitsikov et al. 2023). TRAF7 activation of the MEKK3 pathway enhances JNK and P38 activity, directly potentiating AP1 (activator protein 1) activity and indirectly potentiating CHOP (C/EBP-homologous protein) function. The JNK-AP1 pathway is indeed associated with osteosarcoma development and progression (Papachristou et al. 2003). Overall, these interactions induce expression of multiple genes, including those involved in proliferation and differentiation, cell survival and apoptosis, immunity, and vascular shear stress response (Bouwmeester et al. 2004). One major cascade involving MEKK3 signaling that is upregulated by TRAF7 is depicted centrally in Fig. 2, showing the NF-κβ pathway mounting an immune response to extracellular stimulation via toll-like receptors (TLR), TRAF7, and MEKK3. TRAF7 lacks the TRAF domain and so does not bind to TLRs directly, but, instead, work downstream of both membrane-based and endogenous TLRs.

As part of the MEKK3-MEK5-ERK5 signaling cascade, TRAF7 also upregulates the expression of shear stress-responsive transcription factor Krüppel-like factor 2 (KLF2) and KLF4, essential regulators of vascular physiology (Novodvorsky and Chico 2014). This is important in the response to the shear stress of laminar blood flow, where KLF2 is activated and has multiple vaso-protective effects, including the inhibition of endothelial cell apoptosis, leukocyte adhesion and migration, and platelet aggregation (Paudel et al. 2021; Komaravolu et al. 2014). TRAF7 also plays an important role in the development of vascular endothelial cells as well as in maintaining endothelial integrity (Ihuoma et al. 2025). Both global and endothelial cell-specific knockouts of *TRAF7* in mice were embryonically lethal due to a dysfunction of endothelial integrity, indicating that TRAF7 is essential in the development of blood vessels (Tsitsikov et al. 2023). *TRAF7* alteration in endothelial cells leads to significant downregulation of KLF2 expression and compromised vascular endothelial integrity during a state of normal blood flow. Accordingly, post-natal deletion of *TRAF7* in mice led to the development of intracranial hemorrhages due to impaired endothelial integrity (Tsitsikov et al. 2023).

Additionally, loss-of-function studies of TRAF7 demonstrate that it is a proteostatic regulator of Ras-(Rat sarcoma virus) related GTPases (Fig. 2) (Najm et al. 2021). In the absence of TRAF7 and its regulation of Ras, Ras signaling potentiates the Ras-Raf-MEK1/2-ERK1/2 kinase pathway, leading to upregulation of proliferation-related gene expression. This pathway activation has been shown to promote anchorage-independent growth of a subset of meningiomas, a tumor of the meninges in the central nervous system (CNS) (Najm et al. 2021). Increased TRAF7 activity regulates the degradation of apoptosis signal-regulating kinase-1 phosphorylation and induces cardiac hypertrophy in a mouse model (Che et al. 2024).

### TRAF7-induced ubiquitination

TRAF7 serves an important regulatory role, with ubiquitination being an important aspect of its function. As an E3 ubiquitin ligase, it adds ubiquitin tags to multiple proteins to initiate degradation, changes in localization, or other cellular functions. TRAF7 ubiquitinates NEMO, an essential part of the NF-κβ pathway, with critical roles in inflammation, proliferation, and apoptosis (Kim et al. 2003; Tsikitis et al. 2010). For example, inactive transcription factor NF-κβ complexes with two IkB kinases (IKKα and IKKβ) and NEMO, a regulatory scaffolding protein. TRAF7 polyubiquitinates the Lys-29 region of NEMO, targeting the complex for lysosomal degradation and thereby regulating NF-κβ gene expression (Zotti et al. 2011; Tang et al. 2018). TRAF7 also ubiquitinates p65, resulting in its degradation, as a further step in the regulation of this cascade (Zotti et al. 2011). NF-κβ activation also leads to the expression of deubiquitinating enzymes, including CYLD. CYLD downregulates the inflammatory response by inhibiting NF-κβ activation through the deubiquitination of multiple proteins, including TRAF7, contributing to a mechanism of autoregulatory feedback (Fig. 2) (Yoshida et al. 2005). TRAF7 is thus a positive and negative regulator of the NF-κβ kB pathway via its action on MEKK3 and the NEMO complex, respectively.

Studies with based on cell culture work suggest that TRAF7 is also involved in regulating circadian rhythm by ubiquitination and degradation of D-site binding protein (DBP). DBP is a transcription factor responsible for driving the oscillation of multiple physiological processes throughout the day (Yoshitane et al. 2019). Specifically, TRAF7 controls DBP levels by binding to and tagging the K48 subunit of DBP for polyubiquitination and subsequent lysosomal degradation. TRAF7 upregulation leads to lower DBP levels and shorter period lengths on the cellular clock, (Masuda et al. 2024) suggesting that TRAF7 plays broader role in homeostatic processes like sleep–wake cycles and metabolism. TRAF7 also performs self-ubiquitination, leading to its degradation as an autoregulatory mechanism (Bouwmeester et al. 2004).

### TRAF7-induced SUMOylation

In addition to ubiquitination, TRAF7 also serves other regulatory roles through protein SUMOylation, and post-translational modifications that influence transcription factors (Vertegaal 2022). SUMOylation is the process where small ubiquitin-like modifier (SUMO) proteins bind to the lysine residues of targeted proteins. In contrast to ubiquitination, SUMOylation typically leads to protein localization, stability, or modified activity rather than degradation (Seeler and Dejean 2003). Unlike ubiquitination, SUMOylation is a dynamic and reversible process, and plays a crucial role in regulating the translocation of proteins into and out of the nucleus and sub-nuclear organization (Seeler and Dejean 2003). SUMOylation generally downregulates gene expression, although it can also upregulate expression depending on the gene (Rodriguez et al. 1999).

TRAF7 binds to the DNA-binding domain of the c-Myb proto-oncogene product (c-Myb) with its WD40 domain and tags c-Myb for SUMOylation at Lys-523 and Lys-499. c-Myb is a transcription factor that regulates cell proliferation, playing an important role in hematopoietic, stem, and epithelial cell differentiation. It is also an oncogene overactivated in leukemia, breast cancer, and colorectal cancer (Morita et al. 2005). SUMOylation of c-Myb by TRAF7 sequesters c-Myb in the cytoplasm, limiting translocation to the nucleus and inhibiting its function as a transcription factor.

#### TRAF7-mediated regulation of protein stability

TRAF7 also interacts with the endothelial cell-specific ROBO4, suppressing vascular endothelial growth factor (VEGF)-induced angiogenesis and hyperpermeability (Shirakura, et al. 2019) as well as binding to and stabilizing VE-cadherin at cell junctions. VE-cadherin, an adhesion molecule responsible for maintaining cell–cell adhesion in endothelial cells, is downregulated during tumor necrosis factor α (TNFα)-induced vascular hyperpermeability and inflammatory response, and vascular permeability is maintained by the ROBO4-TRAF7 complex (Fig. 2) (Shirakura, et al. 2019). The TRAF7-ROBO4-VE-cadherin signaling pathway is essential for maintaining blood brain barrier integrity and preventing leakage. In rodents, the ROBO4-TRAF7 complex also suppresses prostaglandin-endoperoxide synthase 2 (PTGS2) expression, suggesting it may protect against PTGS2-associated inflammatory disorders, including arthritis (Tanaka et al. 2024). Additionally, in preclinical models of neuroregeneration, TRAF7 destabilizes hypoxia-inducible factor HIF1a induction that promotes peripheral nerve regeneration, impairing its regenerative function. However, histone deacetylase 8 (HDAC 8) regulates TRAF7 to inhibit its effects on HIF1a and promote nerve recovery (Hertzog et al. 2025). In a rodent spinal cord injury model, TRAF7 was upregulated after injury in neurons along with active caspase-3, leading to apoptosis of neurons (Xu et al. 2018).

Under physiological conditions, TRAF7 participates in diverse processes including immunity, vascular stability, developmental signaling and circadian regulation, reflecting its role as a central regulatory node which helps explain why its mutation/alterations can lead to multi-system disease. Dysregulation of TRAF7 expression can have several different physiological effects depending on multiple factors including mutation site on the TRAF7 gene, the tissue/pathway involved, and age. It is therefore essential to understand how specific molecular changes to TRAF7 manifest clinically.

## TRAF7 alterations and associated conditions

De novo point mutations in the *TRAF7* gene occur in the germline, typically causing developmental disorders, and it can also be somatic, where they are strongly linked to tumor development (Palma-Milla et al. 2024; Tokita et al. 2018). Most reported germline and somatic mutations are hemizygous missense mutations that occur at various locations on the C-terminal end of the protein, most frequently on the WD40 repeats (see Fig. 1) (Zhu et al. 2018). These mutations have a dominant negative effect as they dimerize with the wild type TRAF7 protein, thereby disrupting its function, in contrast to loss-of-function mutations or haploinsufficiency (Palma-Milla et al. 2024). Additionally, most reported germline and somatic mutations are recurrent across patients, either as germline or somatic, without overlap between mutations that occur in the germline versus those that are somatic. The reason somatic and germline mutations are mutually exclusive is unclear. Somatic and germline mutations may induce distinct protein–protein interactions and mechanisms. Somatic mutants may also have a more severe effect in embryonic cells leading to death. Currently, genetic testing for TRAF7 is available for patients with unexplained developmental syndromes or certain tumors, highlighting the clinical relevance of TRAF7 mutations. In this section, we discuss developmental disorders and tumors associated with *TRAF7* alterations and depict many of these alterations in Fig. 1.

### Role of TRAF7 germline mutations in developmental delay.

Both preclinical and clinical work have established that *TRAF7* expression is essential for fetal development (Palma-Milla et al. 2024). In a 60% *TRAF7* knockdown model in zebrafish embryos, reduced TRAF7 expression was observed along with developmental defects in multiple organ systems, including microencephaly, a curved body axis (scoliosis), an unconsumed yolk sac (residual yolk sac), pericardial edema, and short stature when compared to controls (Song et al. 2024). In humans, heterozygous germline mutations have been identified in at least 70 patients with a developmental disorder involving cardiac, facial, and digital anomalies, as well as developmental delay (Palma-Milla et al. 2024). This syndrome in humans was recently termed TRAF7-related cardiac, facial, and digital anomalies with developmental delay (CAFDADD) or TRAF7 syndrome (Palma-Milla et al. 2024; Tokita et al. 2018; Castilla-Vallmanya et al. 2020). There is high phenotypic variability within the population of patients with TRAF7 syndrome, but common developmental and neurological features include intellectual disability, speech delay, motor impairment, congenital heart defects, facial dysmorphism, and skeletal anomalies, while a subset of patients are diagnosed with autism spectrum disorder or epilepsy (Castilla-Vallmanya et al. 2020; Neale et al. 2012; Krumm et al. 2015; Pisan et al. 2024).

TRAF7 syndrome is caused by several pathogenic *TRAF7* mutations, most of which are located within the WD40 repeat protein domain, and the most recurrent of these mutations are p.Arg655Gln, p.Arg524Trp, and p.Phe617Leu (Palma-Milla et al. 2024; Najm et al. 2021; Goode et al. 2018) (see Fig. 1). Fibroblasts obtained from skin biopsies of TRAF7 syndrome patients with germline mutations were evaluated to identify alterations in the expression of other genes in these patients in comparison to controls. Of 76 differentially expressed genes identified in TRAF7 syndrome patients, the main theme identified was dysregulation of human developmental genes, including *FLNB*, *IGFBP7*, *NOTCH3*, *BCL2*, and *PTGS2* (Zotti et al. 2011; Castilla-Vallmanya et al. 2020). Genes implicated in pathways associated with axonal guidance, Wnt/Ca^2+^ signaling, and cardiac hypertrophy, among others, were enriched in *TRAF7* mutant patients with TRAF7 syndrome (Castilla-Vallmanya et al. 2020). It will be important for future work to evaluate the pathophysiological impact of *TRAF7* mutations on these other pathways, which collectively contribute to the phenotype of TRAF7 syndrome.

### Role of TRAF7 somatic mutations in tumorigenesis

Beyond germline mutations associated with TRAF7 syndrome and developmental delays, pathological somatic mutations also occur in *TRAF7*, which are associated with subsets of meningiomas, mesotheliomas, intraneural perineuriomas, and adenomatoid tumors of the genital tract (Yoshida et al. 2005; Zhang et al. 2021; Najm et al. 2021; Goode et al. 2018; Lenartowicz et al. 2023) (see Fig. 1). In fact, TRAF7 mutations are emerging as prognostic molecular biomarkers in certain tumor types. Somatic mutants are also mostly missense mutations, although loss-of-function mutants have also been identified. As described earlier, although there is overlap between somatic mutations across different tumor types, there is no overlap with germline TRAF7 syndromic mutations (Palma-Milla et al. 2024). There are cohorts of patients with these tumors that are enriched for those with *TRAF7* mutations, including 97% of secretory meningiomas (Reuss et al. 2013), 90% of well-differentiated papillary peritoneal mesothelial tumors (Offin et al. 2024; Stevers et al. 2019), and 85% of uterine adenomatoid tumors (Itami et al. 2021). We focus here on the importance of *TRAF7* somatic mutations in meningioma molecular biology, which is well-studied, and outline the importance of TRAF7 as a biomarker in mesothelioma, perineurioma, and adenomatoid tumors.

*TRAF7* somatic mutations are most thoroughly studied in meningiomas, tumors arising from the meninges covering the brain. Meningiomas are stratified into grades 1–3 by World Health Organization criteria, and the majority are benign. Meningiomas account for 35% of all intracranial primary tumors (Wiemels et al. 2010) and approximately 60% of benign CNS tumors (Go and Kim 2023). The most common molecular alteration in meningioma is the loss of one copy of chromosome 22/alteration of the Neurofibromatosis 2 gene (*NF2*), both in sporadic meningiomas and those associated with the syndrome of neurofibromatosis type 2. Mutations in *TRAF7* are the second most common alteration in meningioma, which are found in 25% of tumors (Reuss et al. 2013; Go and Kim 2023). *TRAF7* mutations are exclusive to meningiomas without *NF2* alterations and are enriched in secretory meningioma (Zotti et al. 2017). *TRAF7* mutations in meningioma co-occur either with mutations in Kruppel-like factor 4 (*KLF4*) or in other genes, including v-Akt murine thymoma viral oncogene homolog 1 (*AKT1*) or phosphatidylinositol-4,5-bisphosphate 3-kinase catalytic subunit alpha (*PIK3CA*) variants (Reuss et al. 2013; Clark et al. 2013; Szulzewsky et al. 2024). TRAF7 (along with NF2 and PI3K) associated meningiomas are more aggressive and recur at a significantly higher rate than other molecular subgroups of meningiomas (KLF4, POLR2A, and SMARCB1) (Youngblood et al. 2021). While *TRAF7* mutations are usually de novo, there are some mutations that recur including missense mutations such as the Asn520 variant which has been reported more than 30 times in meningioma patients (Castilla-Vallmanya et al. 2020). Other mutations including p.His521Arg and p.Ser561Arg are highly recurrent in adenomatoid tumors of the genital tract but not in meningiomas.

While *TRAF7* altered meningiomas can occur in any of the locations where meningiomas arise, they are typically located along the anterolateral skull base or the convexities anterior to the coronal suture (Mishra-Gorur et al. 2023; Hua et al. 2024). These alterations are the most frequent ones associated with meningioma-induced hyperostosis, commonly with spheno-orbital meningiomas, although the mechanism underlying this phenomenon is unclear (Umbach et al. 2024). There has been extensive work aiming to elucidate the mechanism of meningioma development in tumors with *TRAF7* alterations. It has been shown that depletion of TRAF7 leads to changes in cytoskeletal organization due to hyperactivation of Ras-related GTPases, including cell division cycle 42 (CDC42), Kirsten Rat Sarcoma Viral Oncogene Homolog (KRAS), and Harvey Rat Sarcoma Viral Oncogene Homolog (HRAS) that interact with TRAF7 via the WD40 domain (Najm et al. 2021). Firstly, mutations in the WD40 domain of the TRAF7 impair its interactions with Ras GTPases in the majority of meningiomas (Pisan et al. 2024; Szulzewsky et al. 2024; Colleran et al. 2023). Secondly, ubiquitination is a crucial process for regulating GTPases, and alteration of this process with *TRAF7* mutation can lead to dysregulation and increased GTPase activity. Additionally, TRAF7 mutants have the ability to heterodimerize with wild-type TRAF7, impairing the normal function of the wild-type protein (Mishra-Gorur et al. 2023) and resulting in either loss or gain of functions depending on the location of the mutation. For instance, mutations in the RING domain that interfere with E3 ligase activity of TRAF7 cause loss of function mutations, while mutations in the WD40 repeats may cause conformational changes that interact with novel proteins leading to a gain of function. Overall, Ras/MAPK signaling activation due to TRAF7 deficiency leads to anchorage-independent growth of meningiomas (Sahm et al. 2025).

Ras activation also leads to upregulation of Semaphorin signaling via a *KLF4*-dependent process that decreases GTPase activity via a negative feedback mechanism. KLF4 is a tumor suppressor and a regulator of growth through the Semaphorin pathway that interacts with TRAF7 through a mechanism that is dependent on S-sulfhydration of TRAF7 by H_2_S at cystine 327 (Najm et al. 2021; Li et al. 2024). S-sulfhydration is a post-translational process to alter TRAF7 structure and promote TRAF7-KLF4 binding. Approximately 40% of *TRAF7* altered meningiomas carry a recurrent loss-of-function *KLF4*-K409Q mutation that impairs this negative feedback mechanism, thereby allowing for the promotion of meningeal cell growth described above (Clark et al. 2013). Overall, *TRAF7* alteration activates both tumor supportive (CDC42) and tumor suppressive (KLF4) pathways, and the co-occurrence of *TRAF7* and *KLF4* mutations allows for hyperactivation of Ras signaling as well as impaired tumor suppression, leading to meningioma growth. Additionally, *AKT1* is co-mutated in *TRAF7-*mutated meningiomas without *KLF4* mutations (Hirano et al. 2024). AKT1 is a serine/threonine-protein kinase that regulates many signaling pathways that are involved in cell growth, motility, metabolism, and proliferation (Alwhaibi et al. 2019). The *AKT1* variant *AKT1*^E17K^ found in meningiomas activates the mTOR signaling pathway, which promotes cell growth, survival, and proliferation while inhibiting apoptosis (Davies et al. 2015; Rascio et al. 2021; John et al. 2022). *PIK3CA* co-mutations are also found in meningiomas with *TRAF7* mutations but without *KLF4* mutations, and PIK3CA is also associated with activation of mTOR signaling, as with *AKT1* alterations. In summary, mTOR pathway activation via *AKT1* or *PIK3CA* mutations promotes cell proliferation and acts together with TRAF7 dysfunction in meningiomas without *KLF4* co-mutations (Davies et al. 2015).

Finally, TRAF7 is also an important biomarker of indolent disease in mesothelioma. Well-differentiated papillary mesothelioma is genetically defined by TRAF7 or CDC42 alterations, which are mutually exclusive, and differentiate them from malignant mesothelioma. This is important clinically as these entities are challenging to differentiate histologically but have very different prognoses, with well-differentiated papillary mesothelioma following an indolent course and malignant mesothelioma having poor outcomes (Stevers et al. 2019). *TRAF7* mutations have also been identified in 60% of intraneural perineuriomas but not in extraneural perineuriomas (Klein et al. 2017). TRAF7 also serves as a biomarker for adenomatoid tumors of the genital tract, which are benign serosal mesothelial tumors with a favorable prognosis, distinguishing them from malignant mesothelial tumors that have a much poorer prognosis (Goode et al. 2018; Itami et al. 2021).

### Impact of altered TRAF7 expression in cancer progression

TRAF7 overexpression can also promote tumor progression by impacting its downstream signaling pathways. The mechanisms leading to increased TRAF7 expression are multifactorial and not well understood, but factors like changes in upstream signaling, mutations, and environmental factors could all contribute to increased TRAF7 activity. In hepatocellular carcinoma (HCC), TRAF7 overexpression is associated with an increased tumor size, higher-grade tumors, and poorer prognosis (Zhang et al. 2021; He et al. 2020). TRAF7 overexpression promotes proteasome-related ubiquitination and degradation of KLF4 as well as P53 (tumor protein 53), a tumor suppressor protein involved in DNA repair, cellular senescence, and apoptosis (Zhang et al. 2021). The loss of P53 impairs these processes that regulate growth and thereby allow for a dysregulated increase in cell proliferation (Wang et al. 2013). Overall, this leads to a TRAF7-facilitated increase in HCC proliferation, invasion, and migration as well as reduced apoptosis, which occurs with TRAF7 overexpression but not when TRAF7 expression is attenuated (Zhang et al. 2021). These studies indicate that TRAF7 facilitates HCC by promoting the degradation of tumor suppressor proteins. In contrast to HCC, TRAF7 downregulation in breast cancers is associated with increased P53 expression, which contributes to tumor progression and poorer outcomes (Zhang et al. 2021). P53 in breast cancer cells is mutated and sequesters in the cytoplasm, indicating a loss of regular function. Loss of TRAF7 restricts the cells’ ability to degrade the mutant p53. This causes increased accumulation and impaired p53 function, which can enhance tumor initiation or progression (Wang et al. 2013). Additionally, TRAF7 overexpression has also been implicated in the pathophysiology of prostate cancer through its effect on Homeobox A5 (HOXA5) (Ye et al. 2023). HOXA5 is a transcription factor with essential roles, including those involved in receptor signaling, cellular differentiation, and angiogenesis (Crooks et al. 1999). HOXA5 has also been shown to upregulate P53, thereby playing a role in tumor suppression. TRAF7 expression is upregulated in prostate cancer cells and mediates ubiquitin-mediated degradation of HOXA5, dysregulating the HOXA5-mediated reduction in cancer cell proliferation and invasion via P53 (John et al. 2022; Raman et al. 2000). Currently, there is a growing interest in further evaluating the ubiquitination of P53 and HOXA5 as potential new therapeutic targets for these tumors.

## Challenges and future directions

There is an emerging appreciation of the critical functions of TRAF7 and its involvement in multiple cellular signaling pathways, including those related to proliferation, inflammation, and apoptosis. A key challenge in preclinical TRAF7 research is the development of animal models that may accurately reflect human conditions associated with altered TRAF7 expression. In mice, Traf7 is necessary and essential for blood vessel integrity, with all prenatal *TRAF7* knockout models being embryonically lethal and adult *TRAF7* knockout models exhibiting intracranial hemorrhages and death within weeks of knockout (Tsitsikov et al. 2023). Consequently, these animals have a severe phenotype that limits the evaluation of TRAF7-driven human conditions, including tumors and syndromic changes. Heterozygous knockouts are rarely sufficient to elicit a phenotype, as a single copy of the gene can produce sufficient TRAF7 for normal function. As such, most TRAF7 basic studies use cell lines and non-mammalian models such as zebrafish. While these models are useful in furthering TRAF7 understanding, they do not adequately capture the number and complexity of TRAF7 interactions seen in humans, especially the effects of point mutations on the *TRAF7* gene. A promising strategy for future research is to explore targeted tissue-specific knockouts or CRISPR-based point mutations in animal models to treat severe phenotypes that mimic TRAF7 associated human diseases. However, there are multiple feedback mechanisms that may limit the impact of TRAF7 alterations alone, including those related to KLF4 described in meningioma (Reuss et al. 2013).

Existing literature reveals multiple potential avenues to explore therapeutic targets that may prevent or treat TRAF7 related human disease in the future. One approach may be to use gene-editing technology such as CRISPR-based systems to target and restore critical TRAF7 function, along with associated alterations like *KLF4* point mutations involved in meningioma. Targeted therapies that aim to inhibit TRAF7-induced ubiquitination of P53 and HOXA5 can be explored as a means to suppress TRAF7-related tumor growth in breast and prostate cancer cells, respectively. While there is no current direct TRAF7 inhibitor, there is also potential for the development of combinatorial treatments that target downstream signaling molecules dysregulated by TRAF7 alteration. For instance, mTOR inhibitors (rapamycin, everolimus) and NK-κB inhibitors (bortezomib, dasatinib) could be used to attenuate pathways downstream of TRAF7 without direct TRAF7 modulation. Further work is needed to comprehensively develop preclinical models of TRAF7-related human disease characterize the molecular landscape of TRAF7-altered tumors and for the evaluation of novel therapeutic options in TRAF7-mediated disease.

## Conclusion

TRAF7, the most recently identified member of the TRAF family of proteins, is a crucial protein involved in a diverse set of essential biological functions. This review outlines its main functions and clinical phenotypes associated with its dysregulation. Physiologically, it acts as an E3 ubiquitin ligase that has a crucial regulatory role in signaling pathways for immune and inflammatory function, cell growth and differentiation, cell survival and apoptosis, and endothelial integrity and function (Table 1). Germline mutations in *TRAF7* lead to the TRAF7 syndrome of developmental delay and other congenital anomalies, while somatic mutations drive tumor development including meningiomas, benign mesotheliomas, intraneural perineuriomas, and adenomatoid tumors of the genital tract (Table 2). *TRAF7* alterations in meningioma co-occur with alterations in *KLF4*, *AKT1*, or *PIK3CA*. *TRAF7* gain-of-function alterations promote progression of tumors in organs such as the liver (hepatocellular carcinoma), breast (breast cancer), and prostate (prostate cancer), distinct from those affected by *TRAF7* mutations, primarily through ubiquitination and degradation of tumor suppressor proteins. We comprehensively describe TRAF7 structure, outline clinical conditions related to TRAF7 alterations, and our current understanding of the underlying pathophysiological mechanisms relating to its impaired function. We show that TRAF7 has a wide range of functions and different subsets of the functions may be altered across a range of TRAF7-associated conditions. Interestingly, this leads to TRAF7 having an oncogene role in some conditions and a tumor suppressor role in others. The E3 ligase activity of TRAF7 can be involved in tumor suppressions by increasing apoptosis and reducing growth. Mutations that diminish E3 ligase activity, as in meningiomas, can lead to increased tumor growth. Additionally, the WD40 domain of TRAF7 is a kinase that promotes growth and proliferation so an increase in its activity can promote tumorigenesis. Further work is needed to clarify the precise mechanisms of TRAF7 that are altered and unaltered in each related condition to guide optimal clinical management. Further work to develop tissue-specific animal models of TRAF7-related disease and enhance our understanding of the molecular vulnerabilities of TRAF7-altered tumors may accelerate the identification of prognostic biomarkers that help guide clinical decision making and targeted therapies for TRAF7-mediated dysfunction.

**Table 1:** Physiological Functions of TRAF7

*TRAF7 Role*	*Model system evaluated*	*Function*	*System/Process Involved*	*Citation(s)*
MAPK signaling pathway regulation	HeLa cells HEK293 cells primary human B cells	Regulates MEKK3-MEK-ERK signaling	NF-κβ activation (immune modulation), JNK activation, AP1 activation, CHOP activation, apoptosis, cell proliferation, differentiation, survival, ubiquitination	Zotti et al. 2012; Bouwmeester et al. 2004; Xu et al. 2004; Zotti et al. 2011; Tang et al. 2018)
Vascular integrity	C57BL/6 mice B6.FVB-tg(Ella-cre)C5379Lmgd/J mice B6.Cg-Tg(Tek-cre)12Flv/J mice C57BL/6-Tg(Cdh5-cre/ERT2)1Rha mice HUVECs HEK293 cells COS-1 cells	SCRIB-TRAF7 signaling via MAPK pathway	Development and maintenance of blood vessel integrity	Tsitsikov et al. 2023; Yoshida et al. 2005; Shirakura, et al. 2019
Immune response	HeLa cells	Regulates NF-κβ signaling through IKKβ/NEMO ubiquitination and MAPK signaling via AP1/JNK-p38 activity	Inflammation, immunity	Bouwmeester et al. 2004; Zotti et al. 2011; Tang et al. 2018
Circadian Period	C57BL/6 mice NIH 3T3 cells HEK293T cells	TRAF7 binding to D-site binding protein	Regulation of circadian rhythm	Masuda et al. 2024
Cellular differentiation	C2C12 mouse myoblasts CV-1 cells HEK293T cells M1 cells DND39 cells	TRAF7 regulates cell cycle exit via NF-κβ and SUMOylates c-Myb for cytoplasmic sequestration	Cell proliferation and differentiation	Tsikitis et al. 2010; Morita et al. 2005
Vascular permeability	129/Sv KO mice HUVECs HEK293 cells COS-7 cells U937 cells	ROBO4-TRAF7 binding prevents TNFα-induced VE-cadherin internalization and RAC1-induced alteration of VE-cadherin	Regulation of permeability and prevention of hyperpermeability	Shirakura, et al. 2019; Tanaka et al. 2024
Peripheral axon regeneration	C57BL/6 J floxed/Cre mice Primary rat Schwann cells HEK293T cells	HDAC8-TRAF7-HIF1α regenerative signaling pathway	Regulates axonal regeneration	Hertzog et al. 2025
Embryonic development	TU and Tg(huC:RFP) Zebra fish	Coiled-Coil region of TRAF7 is essential for fetal development	Developmental defects in multiple organ systems when TRAF7 altered in development	Song et al. 2024

*HUVEC* Human umbilical vein endothelial cells, *IKKβ* Inhibitor of nuclear factor kappa-B kinase subunit beta, *NEMO* NF-kappa-B essential modulator, *MEKK3* Mitogen-activated protein kinase kinase kinase 3, *MEK* Mitogen-activated protein kinase, *ERK*, Extracellular signal-related kinase, *AP-1* Activator protein 1, *JNK* c-Jun N-terminal kinase, *p38* p38 mitogen-activated protein kinase, *CHOP* C/EPB-homologous protein, *c-Myb* MYB proto-oncogene, *VE-cadherin* Vascular endothelial-cadherin, *ROBO4* Roundabout guidance receptor 4, *SCRIB* Scribble planar cell polarity protein, *HDAC8* Histone deacetylase-8, *RAC1* Rac family small GTPase 1, *HIF1α* Hypoxia inducible factor 1 subunit alpha, *KO* Knockout

**Table 2:** Pathophysiological Functions of TRAF7

*Disease*	*Model evaluated*	*Abnormality*	*Clinical features*	*Citation(s)*
Cardiac hypertrophy	C57BL/6 mice *Traf7*-CKO mice Myh6-cre/Esr1 mice *Ask1*-CKO mice neonatal Sprague–Dawley rat cardiomyocytes neonatal rat cardiac fibroblasts	TRAF7-ASK1-JNK-p38-AP1 hypertrophy signaling pathway	Increased TRAF7 activity leads to cardiac hypertrophy	Che et al. 2024
Spinal cord injury (SCI)	Sprague–Dawley rats primary Sprague–Dawley spinal cord neurons	TRAF7 expression upregulated after SCI in neurons	TRAF7 upregulation leads to apoptosis of neurons via active caspase-3 upregulation	Xu et al. 2018
CAFDADD	Patient sample genotyping	*TRAF7* germline mutations: K346E, R371G, S558F, H570D, L595V, T601A, S629N, V646L, R653L, R655Q	Developmental delay Intellectual delay Skeletal, ear, hand, and/or foot anomalies Cardiac defects	Palma-Milla et al. 2024; Tokita et al. 2018; Castilla-Vallmanya et al. 2020; Pisan et al. 2024; Colleran et al. 2023
***Cancer***	***Model evaluated***	***Abnormality***	***Clinical features***	***Citation(s)***
Meningioma	Patient tumor genotyping	*TRAF7* mutations (R153S, C388Y, N520S, H521R, G536S, K615E, or R653Q) co-occurring with *KLF4 (K409Q)*, *AKT1 (E17K)*, or *PIK3CA (E542K, E545K)* alterations in subset of tumors	Enriched in secretory meningiomas without *NF2* mutations Typically located in anterolateral skull base or anterior convexities	Mishra-Gorur et al. 2023; Sahm et al. 2025; Reuss et al. 2013; Najm et al. 2021; Zhu et al. 2018; Go and Kim 2023; Clark et al. 2013; Szulzewsky et al. 2024; Hua et al. 2024; Umbach et al. 2024; Hirano et al. 2024
Mesothelioma	Patient tumor genotyping	*TRAF7* mutations (P398R, N520S, H521R, G536S, Q539H, S561R, Y577C, Y621D) in a subset of tumors	Marker of well-differentiated papillary mesothelioma and not malignant mesothelioma	Offin et al. 2024; Stevers et al. 2019; Itami et al. 2021
Perineurioma	Patient tumor genotyping	*TRAF7* (L519P, H521R, S561R) mutations in a subset of tumors	Marker in 60% of intraneural perineuromas but not in extraneural perineuromas	Zhu et al. 2018; Lenartowicz et al. 2023; Klein et al. 2017
Adenomatoid tumors	Patient tumor genotyping HEK293T cells	*TRAF7* (H521R, S561R) mutations in a subset of tumors	Enriched in benign genital tract adenomatoid tumors and not malignant tumors	Zhu et al. 2018; Goode et al. 2018; Itami et al. 2021
Hepato-cellular carcinoma	Patient tumor genotyping Cell lines: PLC5 cells HepG2 cells MHCC97H cells HEK293T cells HCC cells HCCLM3 cells Huh-7 cells SK-Hep1 cells	*TRAF7* overexpression leads to p53 and KLF4 ubiquitination and degradation	*TRAF7* overexpression is associated with increased tumor size, higher grade, invasion, and poorer prognosis	Zhang et al. 2021; He et al. 2020
Breast cancer	Patient tumor genotyping	TRAF7 downregulation leads to increased p53 expression	TRAF7 downregulation correlated with a poorer prognosis	Wang et al. 2013
Prostate cancer	RWPE-1 cells PCa cells DU145 cells 22RV1 cells PC-3 cells VCap cells Xenograft tumor model	TRAF7 overexpression leads to HOXA5 ubiquitination and degradation	TRAF7 overexpression correlated with tumor proliferation	Ye et al. 2023

*AP-1* Activator protein 1, *JNK* c-Jun N-terminal kinase, *p38* p38 mitogen-activated protein kinase, *HOXA5* homeobox A5, *p53* Tumor protein 53, *VE-cadherin* Vascular endothelial-cadherin, *ROBO4* Roundabout guidance receptor 4, *SCRIB* Scribble planar cell polarity protein, *ASK1* Mitogen-activated protein kinase kinase kinase 5, *CAFDADD* Cardiac, facial, and digital anomalies with developmental delay, *KO* Knockout, *CKO* Conditional knockout

## Data Availability

Data sharing is not applicable to this article as no datasets were generated or analyzed during the current study.

## Abbreviations

TRAF7: TNF receptor associated factor 7
TNF: Tumor necrosis factor
TLR: Toll-like receptor
Ras: Rat sarcoma virus
KLF: Krüppel-like factor
MEK1/2: Mitogen-activated protein kinase 1/2
ERK1/2: Extracellular signal-related kinase 1/2
MEKK3: Mitogen-activated protein kinase kinase kinase 3
MEK5: Mitogen-activated protein kinase 5
ERK5: Extracellular signal-related kinase 5
c-Myb: MYB proto-oncogene
MyD88: Myeloid differentiation primary response 88
IKKα: Inhibitor of nuclear factor kappa-B kinase subunit alpha
IKKβ: Inhibitor of nuclear factor kappa-B kinase subunit beta
NEMO: NF-kappa-B essential modulator
IkBα: Inhibitor of nuclear factor kappa B alpha
NF-κβ: Nuclear factor kappa-light-chain-enhancer of activated B cells
p50: Protein 50 subunit of NF-κB
p65: RELA (RELA Proto-Oncogene, NF-KB Subunit) gene
v-rel: Avian reticuloendotheliosis viral oncogene homolog A
p38: P38 mitogen-activated protein kinase
CYLD: CYLD lysine 63 deubiquitinase
HOXA5: Homeobox A5
p53: Tumor protein 53
JNK1/2: C-Jun N-terminal kinase 1/2
AP-1: Activator protein 1
Ub: Ubiquitin
HUVEC: Human umbilical vein endothelial cells
VE-cadherin: Vascular endothelial-cadherin
ROBO4: Roundabout guidance receptor 4
SCRIB: Scribble planar cell polarity protein
ASK1: Mitogen-activated protein kinase kinase kinase 5
HDAC8: Histone deacetylase-8
RAC1: Rac family small GTPase 1; HIF1α, hypoxia inducible factor 1 subunit alpha
CAFDADD: Cardiac, facial, and digital anomalies with developmental delay; KO, knockout
RTK: Receptor tyrosine kinase
VEGF: Vascular endothelial growth factor
PTGS2: Prostaglandin-endoperoxide synthase 2
PIK3CA: Phosphatidylinositol-4,5-bisphosphate 3-kinase catalytic subunit alpha
AKT1: V-Akt murine thymoma viral oncogene homolog 1
